# Correction: Additive effect of diabetes mellitus on the prevalence and prognosis of sarcopenic obesity: Implications for all-cause mortality

**DOI:** 10.1371/journal.pone.0334531

**Published:** 2025-10-13

**Authors:** Ting-Ju Kuo, Shih-Wei Huang, Hui-Wen Lin

An additional affiliation is missing for the third author. Hui-Wen Lin is also affiliated with the ICF Research Center, Shuang Ho Hospital, Taipei Medical University, Taipei, Taiwan.
