# Supplementary material for: Additive effect of diabetes mellitus on the prevalence and prognosis of sarcopenic obesity: Implications for all-cause mortality
Source: PLoS One. 2025 Jun 10;20(6):e0325423. doi: 10.1371/journal.pone.0325423 (PMC12151415; doi:10.1371/journal.pone.0325423)
Supplement: Attachment — Submitted filename: Response to Reviewers.docx [file pone.0325423.s002.docx]

**Dear Dr. Perna:**

**May 5, 2025**

**Thank you for considering our previously submitted manuscript, titled “**Additive Effect of Diabetes Mellitus on the Prevalence and Prognosis of Sarcopenic Obesity: Implications for All-Cause Mortality.**” We have revised the manuscript in accordance with** comments provided by **you and the reviewers. All questions and concerns have been** thoroughly **addressed in this document. We look forward to your response.**

Sincerely

Hui-Wen Lin, PhD

Department of Mathematics, Soochow University, 70 Linhsi Road, Shihlin, Taipei, Taiwan

Tel: 886-2-2881-9471 ext. 6701

Fax: 886-2-8861-1230

Email: hwlin@scu.edu.tw

ACADEMIC EDITOR: This is an interesting paper, but it needs minor revisions.

**Response: Thank you for your positive feedback and constructive suggestions. We have carefully revised the manuscript in accordance with your comments, and we believe that the revisions have enhanced the clarity and overall quality of the manuscript.**

Specifically, the introduction could benefit from a clearer articulation of the research gap and how the study addresses it.

**Response: We have revised the Introduction section to improve its clarity. The updated text is as follows:
To address the aforementioned gap, we explored the additive effect of DM on mortality in individuals with sarcopenic obesity by using nationally representative data. We also examined cause-specific mortality data to precisely define the risk profile of this population. By elucidating these relationships, our goal was to inform future prevention and intervention strategies targeting individuals affected by the dual burden of DM and sarcopenic obesity. (p. 4-5, lines 86–137)**

Additionally, some sections of the methodology require more detailed explanations to ensure reproducibility.

**Response: We have revised the Materials and Methods section to provide more detailed explanations as follows.**

**Statistical analysis**

**Baseline characteristics were compared in participants in the HA-LM group with and without DM. Continuous variables were compared using weighted t tests or Wilcoxon’s rank-sum tests, as appropriate, and are reported as mean ± standard error. Categorical variables were compared using chi-square tests and are presented as unweighted counts (with weighted percentages). A two-sided p value of <0.05 was considered statistically significant.**

**After a survival analysis was conducted, Kaplan–Meier survival curves were constructed to estimate cumulative survival over time for the two groups (HA-LM with DM vs. HA-LM without DM), with a log-rank test used to compare survival distributions. Time zero was set as the NHANES examination date, and time was measured in months since enrollment.**

**To quantify the association between DM and mortality, Cox proportional-hazards models were employed. Initially, a crude (unadjusted) hazard ratio (HR) was calculated, followed by a multivariable-adjusted HR to control for potential confounders. Covariates were then selected on the basis of subject-matter knowledge and bivariate analyses and included the following: age, sex, race or ethnicity, BMI, hypertension status, SII, albumin, alkaline phosphatase, BUN, creatinine, triglycerides, and total cholesterol. The ASMI and FMI were excluded from the Cox model because all participants had already met the HA-LM phenotype criteria, and their inclusion may introduce multicollinearity with BMI.**

**The proportional-hazards assumption for the Cox model was tested by examining Schoenfeld residuals and log(−log) survival plots for each covariate. No significant violation of the proportional-hazards assumption was observed. Specifically, the Schoenfeld residual tests were nonsignificant for all model covariates (with a threshold of p < 0.05), and the log(−log) plots demonstrated roughly parallel curves for the DM versus non-DM groups, indicating that the HRs were relatively constant over time.**

**A cause-specific mortality analysis was conducted using cumulative incidence functions for each major cause of death, with other causes treated as competing risks. However, given the relatively small number of deaths in certain categories, these results are presented descriptively (percentages) rather than through formal competing risk models.**

**All statistical analyses were conducted using SAS software version 9.4 (SAS Institute, Cary, NC, USA) and STATA version 12.0 (StataCorp, College Station, TX, USA). (pp. 9–11, lines 232–268)**

A few references are outdated and should be replaced with more recent studies to strengthen the literature review.

**Response: We have reviewed the cited literature and replaced several outdated references with more recent and relevant studies to enhance the accuracy and relevance of the manuscript.**

Finally, there are minor grammatical errors and formatting inconsistencies throughout the text that need to be addressed to improve overall readability and alignment with journal guidelines.

**Response: The manuscript has been carefully edited for language and style by Wallace Academic Editing. The attachment is the English editing certificate.**

Once these revisions are made, the paper will be in excellent shape for publication consideration.

**Response: We have carefully revised the manuscript in accordance with the feedback provided, and we hope that the revised version meets the criteria for publication.**

==============================

Reviewers' comments:

Reviewer's Responses to Questions

Comments to the Author

1. Is the manuscript technically sound, and do the data support the conclusions?

The manuscript must describe a technically sound piece of scientific research with data that supports the conclusions. Experiments must have been conducted rigorously, with appropriate controls, replication, and sample sizes. The conclusions must be drawn appropriately based on the data presented.

Reviewer #1: Yes

Reviewer #2: Yes

**Response: Thank you for your positive feedback.**

2. Has the statistical analysis been performed appropriately and rigorously?

Reviewer #1: I Don't Know

Reviewer #2: Yes

**Response: We have revised the Materials and Methods section to provide more detailed explanations as follows.**

**Statistical analysis**

**Baseline characteristics were compared in participants in the HA-LM group with and without DM. Continuous variables were compared using weighted t tests or Wilcoxon’s rank-sum tests, as appropriate, and are reported as mean ± standard error. Categorical variables were compared using chi-square tests and are presented as unweighted counts (with weighted percentages). A two-sided p value of <0.05 was considered statistically significant.**

**After a survival analysis was conducted, Kaplan–Meier survival curves were constructed to estimate cumulative survival over time for the two groups (HA-LM with DM vs. HA-LM without DM), with a log-rank test used to compare survival distributions. Time zero was set as the NHANES examination date, and time was measured in months since enrollment.**

**To quantify the association between DM and mortality, Cox proportional-hazards models were employed. Initially, a crude (unadjusted) hazard ratio (HR) was calculated, followed by a multivariable-adjusted HR to control for potential confounders. Covariates were then selected on the basis of subject-matter knowledge and bivariate analyses and included the following: age, sex, race or ethnicity, BMI, hypertension status, SII, albumin, alkaline phosphatase, BUN, creatinine, triglycerides, and total cholesterol. The ASMI and FMI were excluded from the Cox model because all participants had already met the HA-LM phenotype criteria, and their inclusion may introduce multicollinearity with BMI.**

**The proportional-hazards assumption for the Cox model was tested by examining Schoenfeld residuals and log(−log) survival plots for each covariate. No significant violation of the proportional-hazards assumption was observed. Specifically, the Schoenfeld residual tests were nonsignificant for all model covariates (with a threshold of p < 0.05), and the log(−log) plots demonstrated roughly parallel curves for the DM versus non-DM groups, indicating that the HRs were relatively constant over time.**

**A cause-specific mortality analysis was conducted using cumulative incidence functions for each major cause of death, with other causes treated as competing risks. However, given the relatively small number of deaths in certain categories, these results are presented descriptively (percentages) rather than through formal competing risk models.**

**All statistical analyses were conducted using SAS software version 9.4 (SAS Institute, Cary, NC, USA) and STATA version 12.0 (StataCorp, College Station, TX, USA). (pp. 9–11, lines 232–268)**

3. Have the authors made all data underlying the findings in their manuscript fully available?

The PLOS Data policy requires authors to make all data underlying the findings described in their manuscript fully available without restriction, with rare exception (please refer to the Data Availability Statement in the manuscript PDF file). The data should be provided as part of the manuscript or its supporting information, or deposited to a public repository. For example, in addition to summary statistics, the data points behind means, medians and variance measures should be available. If there are restrictions on publicly sharing data—e.g. participant privacy or use of data from a third party—those must be specified.

Reviewer #1: Yes

Reviewer #2: Yes

**Response: Thank you for your positive feedback.**

4. Is the manuscript presented in an intelligible fashion and written in standard English?

PLOS ONE does not copyedit accepted manuscripts, so the language in submitted articles must be clear, correct, and unambiguous. Any typographical or grammatical errors should be corrected at revision, so please note any specific errors here.

Reviewer #1: Yes

Reviewer #2: Yes

**Response: Thank you for your positive comment.**

5. Review Comments to the Author

Please use the space provided to explain your answers to the questions above. You may also include additional comments for the author, including concerns about dual publication, research ethics, or publication ethics. (Please upload your review as an attachment if it exceeds 20,000 characters)

Reviewer #1: This manuscript addresses a clinically relevant and timely topic, particularly given the increasing interest in the intersection between diabetes, sarcopenia, and obesity. The use of a large, representative dataset adds strength to the findings. However, some methodological and presentation aspects require revision to improve clarity and scientific rigor. Language and readability:

1-

The manuscript is generally understandable; however, a careful language review by a native English speaker is recommended to improve fluency and ensure a smoother reading experience.

**Response: The manuscript has been carefully edited for language and style by Wallace Academic Editing. The attachment is the English editing certificate.**

2-

Figure 1 – Kaplan-Meier plot:

In Figure 1, not all survival curves are clearly identified or labeled, which may lead to confusion in interpreting the results. I suggest simplifying the figure by displaying only the two curves that directly reflect the study’s primary analysis. This would enhance clarity and focus the reader’s attention on the most relevant comparison.

**Response: We agree that simplifying the figure would improve clarity and focus. In response, we have revised Fig. 1 to present only the two survival curves most relevant to the primary analysis. We believe this adjustment enhances the figure’s readability and more effectively underscores the key findings of our study. (p. 27)**

3

References:

The references listed in the manuscript are inadequate and do not conform to the formatting standards required by PLOS ONE. A thorough revision of the reference section is recommended, ensuring consistency with Vancouver style, including proper citation of journal titles, publication years, volumes, issues, and page numbers.

**Response: We have thoroughly revised the references section to ensure that all citations adhere to the Vancouver style, as required by *PLoS ONE*.**

Reviewer #2: Summary

This paper examined the effects of diabetes mellitus and sarcopenic obesity on mortality, using a sample of U.S. NHANES 2011-2012 to 2017-2018. This study found that individuals with high adiposity and low muscle mass and diabetes mellitus had a higher risk of mortality than those with high adiposity and low muscle mass but without diabetes mellitus. While these findings are novel, and highlight the role of diabetes mellitus on mortality, some areas of the methods require clarification. In particular, there is no detail on the sample (e.g. age range), and weighting of analyses, that are necessary to fully understand both the validity and representativeness of the findings. Please see comments below.

Major Comments

1. No age range is mentioned in the methods; however, only individuals aged 8-59y are eligible to be administered DXA scans in NHANES cycles 2011-2012. It can be surmised that this is the maximum range, but there needs to be an explanation of the age range of eligible participants and what specific age cut-offs were used. Relatedly, trom Table 1 it appears that the average age of the sample is 37y (no DM) and 47y (with DM), making this a somewhat younger sample overall to assess sarcopenic obesity. The sample age range should be made explicit, as this has implications for both the generalizability of the findings, and reporting of other descriptives (e.g. BMI) in the study. A drop-down table showing how the analytic sample was derived from the initial sample would be appropriate for this work.

**Response: We have clarified the age range criteria in the Methods section of the manuscript. Although DXA data are available for NHANES participants aged 8–59 years from 2011 to 2018, our study focused on the adult population. Therefore, we restricted our analysis to adults aged 18–59 years. This is now explicitly stated in the revised manuscript as follows:**

**Although DXA scans are available for NHANES participants aged 8 to 59 years, we restricted our analysis to adults aged 18 to 59 years to focus on the adult population and their conditions. (p. 7, lines 185-187)**

2. Considering that the majority of the initial sample were excluded due to missing data a sensitivity analysis might be warranted. It is unclear how the age group selection may have facto

**Response:** **We acknowledge the importance of understanding the potential impact of sample exclusions on the study findings.**

**Participants were excluded from the analytical sample for common reasons encountered in secondary data analysis, including missing diabetes status, lack of mortality linkage eligibility, and missing covariate data. These exclusions were necessary to ensure the completeness and validity of our models.**

**To address concerns regarding potential bias introduced by these exclusions, we examined the systematic differences in basic characteristics, such as age, sex, and race or ethnicity, between the included and excluded participants. The results revealed no significant differences in these key characteristics between the included and excluded participants, suggesting that the exclusions did not introduce significant bias or affect the generalizability of our findings.**

**We have added this clarification to the Materials and Methods section of the revised manuscript as follows:**

**Participants were excluded if they had missing data on diabetes status, were ineligible for mortality linkage, or had incomplete covariate information—standard exclusions in secondary data analysis to ensure analytical validity. To evaluate the potential for selection bias, we compared key characteristics (e.g., age, sex, and race or ethnicity) in individuals included in the final analytic sample and those excluded. These groups exhibited similar distributions across these characteristics, indicating that the exclusions may not have introduced meaningful bias or compromised the generalizability of our findings. (p. 7, lines 187–194)**
